# The *Eucalyptus* Cuticular Waxes Contribute in Preformed Defense Against *Austropuccinia psidii*

**DOI:** 10.3389/fpls.2018.01978

**Published:** 2019-01-09

**Authors:** Isaneli Batista dos Santos, Mariana da Silva Lopes, Andressa Peres Bini, Bruno Augusto Prohmann Tschoeke, Bruna Aparecida Wruck Verssani, Everthon Fernandes Figueredo, Thais Regiani Cataldi, João Paulo Rodrigues Marques, Luciana Duque Silva, Carlos Alberto Labate, Maria Carolina Quecine

**Affiliations:** ^1^Departament of Genetics, “Luiz de Queiroz” College of Agriculture, University of São Paulo, Piracicaba, Brazil; ^2^Departament of Exact Sciences, “Luiz de Queiroz” College of Agriculture, University of São Paulo, Piracicaba, Brazil; ^3^Departament of Phytopathology, “Luiz de Queiroz” College of Agriculture, University of São Paulo, Piracicaba, Brazil; ^4^Departament of Forest Science, “Luiz de Queiroz” College of Agriculture, University of São Paulo, Piracicaba, Brazil

**Keywords:** cuticular waxes, GC-TOF-MS, preformed defense, rust, susceptibility

## Abstract

*Austropuccinia psidii*, the causal agent of myrtle rust, is a biotrophic pathogen whose growth and development depends on the host tissues. The uredospores of *A. psidii* infect *Eucalyptus* by engaging in close contact with the host surface and interacting with the leaf cuticle that provides important chemical and physical signals to trigger the infection process. In this study, the cuticular waxes of *Eucalyptus* spp. were analyzed to determine their composition or structure and correlation with susceptibility/resistance to *A. psidii*. Twenty-one *Eucalyptus* spp. in the field were classified as resistant or susceptible. The resistance/susceptibility level of six *Eucalyptus* spp. were validated in controlled conditions using qPCR, revealing that the pathogen can germinate on the eucalyptus surface of some species without multiplying in the host. CG-TOF-MS analysis detected 26 compounds in the *Eucalyptus* spp. cuticle and led to the discovery of the role of hexadecanoic acid in the susceptibility of *Eucalyptus grandis* and *Eucalyptus phaeotricha* to *A. psidii*. We characterized the epicuticular wax morphology of the six previously selected *Eucalyptus* spp. using scanning electron microscopy and observed different behavior in *A. psidii* germination during host infection. It was found a correlation of epicuticular morphology on the resistance to *A. psidii*. However, in this study, we provide the first report of considerable interspecific variation in *Eucalyptus* spp. on the susceptibility to *A. psidii* and its correlation with cuticular waxes chemical compounds that seem to play a synergistic role as a preformed defense mechanism.

## Introduction

*Austropuccinia psidii* (G. Winter) Beenken (syn. *Puccinia psidii*) is a basidiomycete that belongs to the Pucciniales order and is the causal agent of myrtle rust (Miranda et al., 2013; Beenken, 2017). The pathogen infects approximately 460 species in 73 genera of the Myrtaceae (Roux et al., 2013; Giblin and Carnegie, 2014; Beenken, 2017). Its hosts include *Eucalyptus* spp. that are important to the forestry industry (Coutinho et al., 1998). Different degrees of severity are observed in *Eucalyptus*, and the disease occurs primarily in young trees and infects actively growing leaves, shoots, inflorescences and apical buds (Glen et al., 2007). The disease is characterized by the production of yellow and pulverulent pustules containing the uredospores that cause the deformation of the leaves, intense defoliation of the branches, stunted growth and even the death of the plants (Ferreira, 1981). Despite the importance of myrtle rust, the mechanisms of resistance of *Eucalyptus* spp. to *A. psidii* remain unclear. The host penetration by rust pathogens occurs preferentially through stomata; however, *A. psidii* penetrates inner *Eucalyptus* tissues through the cuticle and epidermis following the formation of appressoria (Xavier et al., 2001).

Leaf cuticular waxes cover all the aerial plant surfaces and have a multifunctional role, serving as an interface between the plant and biotic or abiotic stresses; its composition includes a polymeric skeleton of cutin covalently attached to a type of lipid known as waxes (Koch et al., 2010; Yeats and Rose, 2013). Plant cuticles are known to be primary barriers against herbivores and pathogens that perform a key factor in the adaptation and evolution of terrestrial plants and that the cutin monomers released are signaling molecules for both the pathogens and plants (Tucker et al., 2010; Serrano et al., 2014). Leaf surface cuticles have been described to be a source of signals that influence the germination and specificity of the host to biotrophic pathogens (Lazniewska et al., 2012). Thus, the effect of cuticular waxes on pathogen germination has been examined by several authors (Kolattukudy et al., 1995; Inuyang et al., 1999; Reisige et al., 2006; Zabka et al., 2007; Hansjakob et al., 2011; Zabka et al., 2014). These extracts obtained from the leaf surface can induce up to 50% germination and appressorium production by pathogenic fungi (Reisige et al., 2006). A long-chain hydrocarbon with a 436 MW (C_31_H_64_) known as hentriacontane was characterized from an active fraction of *Syzygium jambos* leaf wax extract. The active fraction induced up to 88% of uredospore germination and was determined to be involved with the stimulation of rust uredospore germination (Tessmann and Dianese, 2002).

Due to the unique characteristics of the *Eucalyptus* cuticle, our hypothesis supports the concept that the preformed mechanism plays an important role in *Eucalyptus* resistance. Thus, we selected *Eucalyptus* spp. with varying responses to rust myrtle infection in the field and validated the results in controlled conditions. The leaf cuticular wax composition of the six eucalyptus species was characterized using gas chromatography coupled to mass spectrometry (GC-TOF-MS). We also assessed the uredospores germination rate of *A. psidii* exposed to cuticular waxes and specific metabolites identified in susceptible and resistant *Eucalyptus* spp. The morphology of the *Eucalyptus* spp. cuticle was observed using electron microscopy. The polyphasic analysis of the leaf cuticle greatly contributed to a better understanding of the dynamics of the early infection process of *A. psidii* on *Eucalyptus* spp.

## Materials and Methods

### Susceptibility of *Eucalyptus* spp. to *A. psidii*: Field Inference

Natural *Eucalyptus* infection by *A. psidii* was evaluated in young trees up to 2 years old. The trees were located at the Anhembi Experimental Station of Forestry Sciences (EECFA), Anhembi, São Paulo, Brazil (22°40′S and 48°10′W). The disease scores representing the percentage of leaves with rust symptoms were based on the Takahashi (2002) scale modified by Zamprogno et al. (2008): S0 (no pustules or healthy plants); S1 (isolated punctate pustules on the limbs and young leaves); S2 (rust pustules generally sparse or occasionally abundant on the limbs and young leaves), and S3 (pustules abundant on the limbs, petioles and leaves, at the tips of branches and the primary stem and apical necrosis). The index disease (ID) was calculated as described by McKinney (1923). The experiment was a randomized design with 21 genotypes (19 species and 2 hybrids) and 72 biological replicates per genotype. Each replicate consisted of one tree.

After the field assessment, species with enough plantlets to further assays: susceptible (*Eucalyptus grandis* and *Eucalyptus phaeotricha*) and resistant (*Eucalyptus urophylla*, *Eucalyptus camaldulensis*, *Eucalyptus urograndis* and *Eucalyptus robusta*) were selected to validate the data in controlled conditions as described by Leite et al. (2013) with modifications (Quecine et al., 2016). Plantlets of each species were grown under greenhouse conditions for 120 days and transferred to a controlled growth chamber under a 12 h photoperiod (200 μmol m^-1^ s^-1^) at 20°C for acclimatization for 7 days. A suspension of *A. psidii* MF-1 containing 10^5^ uredospores mL^-1^ and 0.05% Tween 20 was sprayed onto the plants. The plants were enclosed in transparent plastic bags for the first 48 h with the first 24 h in complete darkness at 20°C to enable the fungus to germinate. The plants were returned to the growth conditions previously described. The symptoms were assessed in the 3rd–14th day after inoculation based on the scale adapted from Zamprogno et al. (2008). The experiment was conducted in a completely randomized design.

### *A. psidii* Quantification by qPCR in Contrasting *Eucalyptus* Species

The leaves from *Eucalyptus grandis*, *Eucalyptus phaeotricha*, *Eucalyptus urophylla*, *Eucalyptus camaldulensis*, *Eucalyptus urograndis* and *Eucalyptus robusta* were harvested at: 0 hour post inoculation (h.p.i) (control treatment); 72 h.p.i (start of the colonization phase—susceptible species, and no detection of the pathogen—resistant species); 144 h.p.i: colonization of mesophyll cells (susceptible species) and no detection of the pathogen (resistant species); and 336 h.p.i: pustules developed (susceptible species) and no detection of the pathogen (resistant species) based on an assay previously described (Bini et al., 2018). At each time, leaves from the first two pairs of five plantlets per species were collected, immediately frozen in liquid nitrogen and stored at -80°C. Leaves of the control plants were collected at 0 h.a.i. The DNA was extracted from 100 mg of leaves using a DNeasy Plant Mini Kit (Qiagen) according to the manufacturer’s instructions.

The qPCR (Real-Time Quantitative PCR) reaction was performed using an iCycleiQ Real-Time PCR Detection System (BioRad) in a final volume of 25 μL. The *A. psidii* genomic DNA quantification in the *Eucalyptus* spp. leaves was based on an IGS region described by Bini et al. (2018). The *A. psidii* MF-1 DNA serial dilution was performed in triplicate, and the time-course assay samples were generated in duplicates of all five biological replicates per sampled time.

### *Eucalyptus* spp. Cuticular Wax Extraction

Cuticular wax from the young leaves of *Eucalyptus grandis*, *Eucalyptus phaeotricha*, *Eucalyptus urophylla*, *Eucalyptus camaldulensis*, *Eucalyptus urograndis* and *Eucalyptus robusta* was extracted as described by Viana et al. (2010) modified by Bini (2016). One milligram of wax was obtained by immersing and gentle agitating the leaves in 5 mL of chloroform (JT Baker) for 30 s and vacuum concentration (SpeedVac-Eppendorf) for 20 min. Each replicate consisted of six to eight young leaves from one plantlet. Four plantlets were used for each species.

### Chemical Characterization of *Eucalyptus* spp. Cuticular Waxes Using CG-TOF-MS

The GC-TOF-MS (Gas Chromatography coupled to Time-Of-Flight Mass Spectrometry) analysis was performed immediately after the wax extraction. The samples were derivatized as described by Hoffman et al. (2010) with 30 μL of methoxyamine hydrochloride (15 mg mL^-1^) in pyridine for 16 h at room temperature in the dark. The samples were trimethylsilylated by adding 30 μL of *N*-methyl-*N*-(trimethylsilyl) trifluoroacetamide (MSTFA) containing 1% trimethylchlorosilane (TMCS) and incubating the mixture at room temperature for 1 h. Thirty microliters of heptane was added after silylation. Stable isotope reference compounds [1 mg mL^-1^each of (^13^C_3_)—myristic acid, (^13^C_4_)—palmitic acid and (^2^H_4_)—succinic acid] were added to the samples prior to derivatization and used as external standards for quality control. The derivatized samples were analyzed as described by Gullberg et al. (2004). Blank control samples and a series of *n*-alkanes (C12–C40) were also used to obtain the retention indices (Schauer et al., 2005).

One microliter of each derivatized sample was injected splitless into a gas chromatograph 7890A (Agilent Technologies, Santa Clara, United States) coupled with a Comb-xt Autosampler (Leap Technologies, Carrboro, United States). The injector temperature was 280°C; the septum purge flow rate was 20 mL min^-1^, and the purge was turned on after 60 s. The gas flow rate through the column was 1 mL min^-1^. The column used for the GC × GC separation was a DB-5 (20m × 0.18 mm × 0.18 μm; Agilent Technologies, Santa Clara, United States) as the first-dimension column and a RTX-17 (0.9m × 0.10 mm × 0.10 μm; Restek Corporation, U.S., Bellefonte) for the second-dimension column. The column temperature was held at 80°C for 2 min, increased by 10°C min^-1^ to 305°C and held for 10 min. The column effluent was introduced into the ion source of a GC × GC/TOFMS (Pegasus 4D, Leco Corp., St. Joseph, United States). The transfer line and the ion source temperatures were 280 and 250°C, respectively. The ions were generated using a 70 eV electron beam at an ionization current of 2.0 mA, and 10 spectra/s were recorded in the mass range m/z 45–800.

ChromaTOF software v. 4.51 (Leco Corp., St. Joseph, United States) was used to correct the baseline and export all the MS files into a NetCDF format. Peak detection, retention time alignment and library matching were performed using the Target Search package (Cuadros-Inostroza et al., 2009). Metabolites were identified by comparing their retention indexes (±2 s) and spectra (similarity > 600) against the compounds stored in the Golm-Metabolome-Database^1^ (Kopka et al., 2005). Metabolite intensities were normalized using dry weight and total ion chromatogram (TIC).

### Influence of Cuticular Wax on *A. psidii* Germination

The cuticular waxes from *Eucalyptus grandis*, *Eucalyptus phaeotricha*, *Eucalyptus urophylla*, *Eucalyptus camaldulensis*, *Eucalyptus urograndis* and *Eucalyptus robusta* were obtained as described above. One milligram of extracted waxes was diluted in 1 mL of dichloromethane (JT Baker) to remove chloroform residues and vacuum concentrated (SpeedVac-Eppendorf). The waxes were solubilized in 1 mL of dichloromethane (JT Baker) and sonicated for 10 min. This final solution was used in the germination assay. The uredospore solution of *A. psidii* MF-1 was prepared separately in mineral oil (Sigma Aldrich) (10^5^ uredospores per Petri dish), which was mixed with 20 ppm of the cuticle extracts (Tessmann and Dianese, 2002). The uredospore solution was inoculated in Petri dishes containing solid water agar medium (8 g L^-1^) amended with mineral oil and cuticle extracts and incubated for 24 h at 20°C in the dark. Five hundred uredospores were observed for each treatment per replicate, and the germination rate was calculated based on the number of uredospores with a germ tube. The experiment was conducted in three randomized blocks. Water, mineral oil and dichloromethane were considered to be the controls. Uredospore germination was observed using a light microscope (Aziophot) with a digital coupled camera (Zeiss).

### Hexadecanoic Acid Effects on *A. psidii* Germination

To validate the results obtained using GC-TOF-MS, a bioassay was performed using commercial palmitic acid (hexadecanoic acid) (Sigma Aldrich) and a uredospore solution of *A. psidii* MF-1 (10^5^ per Petri dish) diluted in mineral oil (Sigma Aldrich). The palmitic acid was diluted in dichloromethane (JT Baker) at concentrations of 0 (control), 0.5, 2.5, 5.0, 10 and 20 ppm and combined with the solution of uredospores before inoculation in Petri dishes with solid water agar medium (8 g L^-1^) and incubation for 24 h at 20°C in the dark. Germination rates were obtained as described above. The experiment was conducted in a randomized block design with six hexadecanoic acid concentrations using three replicates (Petri dishes) per treatment.

### Scanning Electron Microscopy

We examined the morphology of the epicuticle wax morphology by evaluating the middle third adaxial leaf surface of non-inoculated and inoculated (144 h.p.i.) plantlets of *Eucalyptus grandis*, *Eucalyptus phaeotricha*, *Eucalyptus urophylla*, *Eucalyptus camaldulensis*, *Eucalyptus urograndis* and *Eucalyptus robusta*. The leaves were sampled and fixed in Karnovsky solutions (Karnovsky, 1965), washed in 0.1 M phosphate buffer and post fixed in 1% osmium tetroxide in 0.1 M phosphate buffer (pH 7.2). The samples were washed three times in distilled water and then dehydrated in graded acetones (10% 1×, 30% 1×, 50% 1×, 70% 1×, 90% 1×, 100% 2×, for 15 min each). All these steps were performed at room temperature. The samples were dried to their critical point (Horridge and Tamm, 1969), glued on aluminum stubs and sputter coated with gold. The samples were examined at 20 kV using an LEO VP435 (Zeiss, Oberkochen, Germany) scanning electron microscope.

### Statistical Analysis

The profile of the metabolites from the cuticular waxes was submitted to multivariate analysis to visualize the separation among the *Eucalyptus* spp. using the program Metaboanalyst^2^. Principal component analysis (PCA) was also utilized to identify the tendency of group separation, and an ANOVA (Analysis of variance) was used to identify differentially abundant metabolites among the groups (*p* < 0.05), followed by a comparison test of Scott-Knott averages (*p* < 0.05) (Chong et al., 2018). The results of fungal quantification by qPCR. The germination rate of *A. psidii* in different cuticular waxes and hexadecanoic acid concentrations were submitted to an ANOVA, followed by Scott-Knott’s our Tukey tests (*p* < 0.05) using software R (version 3.4.1).

## Results

### Evaluation of the *Eucalyptus* spp. Rust Susceptibility

Among the 19 species and two hybrids evaluated in the field, just two species were classified as S0—high level of resistance to the pathogen (*Eucalyptus resinifera* and *Eucalyptus toreliodora*); four species were classified as susceptible to *A. psidii*, i.e., more than 50% of the individuals were categorized in the S1, S2 and S3 scale (*Eucalyptus botryoides*, *Eucalyptus deglupta*, *Eucalyptus grandis* and *Eucalyptus phaeotricha*) with IDs ranging from 40.51 to 56.30%. Other species demonstrated a low level of resistance, and more than 50% of the individuals were asymptomatic to rust (ID: 0.64–18.65%) (Table 1).

**Table 1:** Susceptibility level of *Eucalyptus* spp. to *A. psidii*.

Species	Susceptibility scale (%)^a^				Classification^b^	ID (%)^c^
			
	S0	S1	S2	S3		
*Eucalyptus botryoides*	44.44	20.83	26.39	8.33	Susceptible	45.65
*Eucalyptus brassiana*	91.67	1.39	2.78	4.17	Low resistance	9.00
*Eucalyptus camaldulensis*	70.83	19.44	8.33	1.39	Low resistance	18.65
*Eucalyptus cloeziana*	75.00	11.11	12.50	1.39	Low resistance	18.65
*Eucalyptus deglupta*	34.72	19.44	34.72	11.11	Susceptible	56.58
*Eucalyptus dunni*	72.22	20.83	5.56	1.39	Low resistance	16.72
*Eucalyptus exserta*	98.61	1.39	0.00	0.00	Low resistance	0.64
*Eucalyptus grandis*	47.22	25.00	20.83	6.94	Susceptible	40.51
*Eucalyptus microcorys*	83.33	12.50	2.78	1.39	Low resistance	10.29
*Eucalyptus paniculata*	91.67	5.56	1.39	1.39	Low resistance	5.79
*Eucalyptus pellita*	93.06	2.78	2.78	1.39	Low resistance	5.79
*Eucalyptus phaeotricha*	45.83	26.39	4.17	23.61	Susceptible	48.87
*Eucalyptus pilularis*	75.00	19.44	1.39	4.17	Low resistance	16.08
*Eucalyptus propínqua*	94.44	2.78	1.39	1.39	Low resistance	4.50
*Eucalyptus resinifera*	100.00	0.00	0.00	0.00	High resistance	0.00
*Eucalyptus robusta*	97.22	0.00	0.00	2.78	Low resistance	3.86
*Eucalyptus saligna*	65.28	31.94	2.78	0.00	Low resistance	17.36
*Eucalyptus tereticornis*	87.50	6.94	4.17	1.39	Low resistance	9.00
*Eucalyptus toreliodora*	100.00	0.00	0.00	0.00	High resistance	0.00
*Eucalyptus urograndis*	83.33	11.11	5.56	0.00	Low resistance	10.29
*Eucalyptus urophylla*	93.06	4.17	1.39	1.39	Low resistance	5.14


*^*a*^Susceptibility scale adapted from Zamprogno et al. (2008); S0, hypersensitive and/or immune response; S1–S3, detection of pustules with different sporulation intensities. The experiment was a randomized design with 21 genotypes (19 species and two hybrids) and 72 replicates. ^*b*^Susceptible: the total susceptibility scale of S1, S2, and S3 is greater than 50%; low resistance, the total susceptibility scale of S0 is greater than 50%, and high resistance: the total susceptibility scale of S0 is the same as 100%. ^*c*^ID, disease index was calculated as described by McKinney (1923).*

**FIGURE 1:**
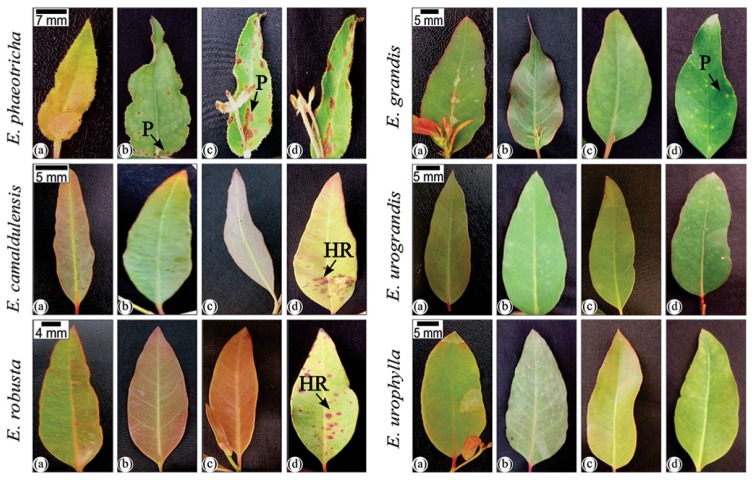
Infection process of *A. psidii* (MF1) in *Eucalyptus* spp. in controlled conditions. Legend: (a): 0 h.p.i. (hours post inoculation); (b): 72 h.p.i.; (c): 144 h.p.i.; (d): 336 h.p.i. Pustules in symptomatic leaves from *Eucalyptus phaeotrica* and *Eucalyptus grandis* are indicated by black arrows. The hypersensitive response in *Eucalyptus camaldulensis* and *Eucalyptus robusta* leaves is indicated by white arrows.

The susceptible/resistance level of the *Eucalyptus* spp. against *A. psidii* was validated in controlled condition: *Eucalyptus urophylla*, *Eucalyptus camaldulensis*, *Eucalyptus robusta* and *Eucalyptus urograndis* were classified as S0, i.e., resistant to *A. psidii* in these conditions with a hypersensitive response in *Eucalyptus camaldulensis* and *Eucalyptus robusta* at 336 h.p.i. The susceptibility of *Eucalyptus grandis* and *Eucalyptus phaeotricha* was confirmed by visualization of the typical symptoms of rust: chlorotic stains which turned into pustules, resulting in a mass of uredospores. The symptoms on the *Eucalyptus phaeotricha* leaves occurred earlier compared to those on *Eucalyptus grandis* (Figure 1).

The infection was also monitored over time by the temporal quantification of *A. psidii* in the leaf tissues of *Eucalyptus grandis*, *Eucalyptus phaeotricha*, *Eucalyptus urophylla*, *Eucalyptus camaldulensis*, *Eucalyptus urograndis* and *Eucalyptus robusta* using qPCR. *A. psidii* was not detected in non-inoculated control samples. The pathogen was detected up to 72 h.p.i in all the species inoculated with *A. psidii* uredospores ranging between 0.04 (*Eucalyptus camaldulensis*) to 0.95 pg (*Eucalyptus phaeotricha*). Non-pathogenicity was detected at 144 and 336 h.p.i. in the resistant species (*Eucalyptus urophylla*, *Eucalyptus camaldulensis*, *Eucalyptus urograndis* and *Eucalyptus robusta*) (Table 2).

**Table 2:** Quantification of *A. psidii* (MF1) during *Eucalyptus* spp. infection.

Species	*A. psidii* quantification (DNA pg)^∗^			
	
	0 h.a.i	72 h.a.i	144 h.a.i	336 h.a.i
*Eucalyptus grandis*	0.21 (±0.09)^b^	0.28 (±0.16)^b^	0.58 (±0.42)^b^	2.71 (±1.31)^a^
*Eucalyptus phaeotricha*	0.42 (±0.09)^c^	0.95 (±0.41)^c^	6.75 (±3.43)^b^	11.8 (±6.53)^a^
*Eucalyptus urograndis*	0.51 (±0.12)^a^	0.14 (±0.08)^b^	0.00 (±0.00)^c^	0.00 (±0.00)^c^
*Eucalyptus urophylla*	0.05 (±0.04)^b^	0.29 (±0.13)^a^	0.00 (±0.00)^c^	0.00 (±0.00)^c^
*Eucalyptus robusta*	0.20 (±0.13)^a^	0.04 (±0.04)^a^	0.00 (±0.00)^b^	0.00 (±0.00)^b^
*Eucalyptus camaldulensis*	0.04 (±0.04)^b^	0.21 (±0.12)^a^	0.00 (±0.00)^c^	0.00 (±0.00)^c^


*^∗^*A. psidii* (MF1) quantification in *Eucalyptus* spp. was measured in pg of the pathogen DNA present in 5 μg of total DNA from infected leaves. The average of five biological replicates was determined at each sampling time; values between the parentheses represent the SE. ^*a–c*^Values with the same letter within a line are not significantly (*p* > 0.05) different according to Tukey’s test.*

### *Eucalyptus* spp. Cuticle Wax Characterization

**FIGURE 2:**
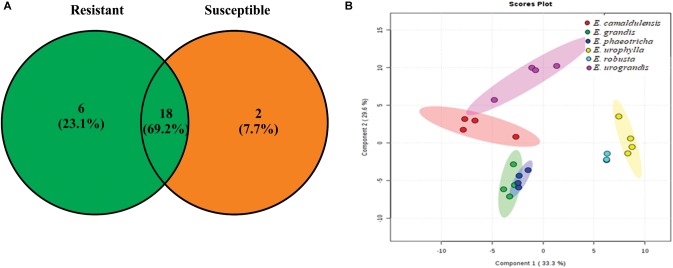
Metabolic profile from the cuticle waxes of susceptible (*Eucalyptus grandis* and *Eucalyptus phaeotricha*) and resistant (*Eucalyptus camaldulensis*, *Eucalyptus urophylla*, *Eucalyptus robusta* and *Eucalyptus urograndis*) *Eucalyptus* species against *A. psidii*. **(A)** The Venn diagram shows the number of overlapping metabolites among the resistant (R) and susceptible (S) treatments. **(B)** Principal component analysis (PCA) of the metabolic profile of cuticle *Eucalyptus* spp. in which susceptible species (*Eucalyptus grandis* and *Eucalyptus phaeotricha*) are represented by green and blue, respectively, and the resistant plants (*Eucalyptus camaldulensis*, *Eucalyptus urophylla*, *Eucalyptus robusta* and *Eucalyptus urograndis*) are shown in red, yellow, light blue and pink colors, respectively.

GC-TOF-MS analysis enabled the identification of 26 metabolites present in the cuticular waxes from the *Eucalyptus* spp. Two metabolites of the 26 compounds were only identified in the susceptible species of *Eucalyptus*, and six were exclusive to resistant *Eucalyptus* species (Figure 2A). The total metabolites obtained from the cuticular waxes were submitted to multivariate analysis to visualize the separation among the treatments in relation to the metabolic content. The PCA revealed a clear tendency of the clustering among susceptible (*Eucalyptus grandis* and *Eucalyptus phaeotricha*) and resistant (*Eucalyptus camaldulensis*, *Eucalyptus urograndis*, *Eucalyptus robusta* and *Eucalyptus urophylla*) species. The sum of the first two principal components explained 54.8% of the total variance (Figure 2B).

**FIGURE 3:**
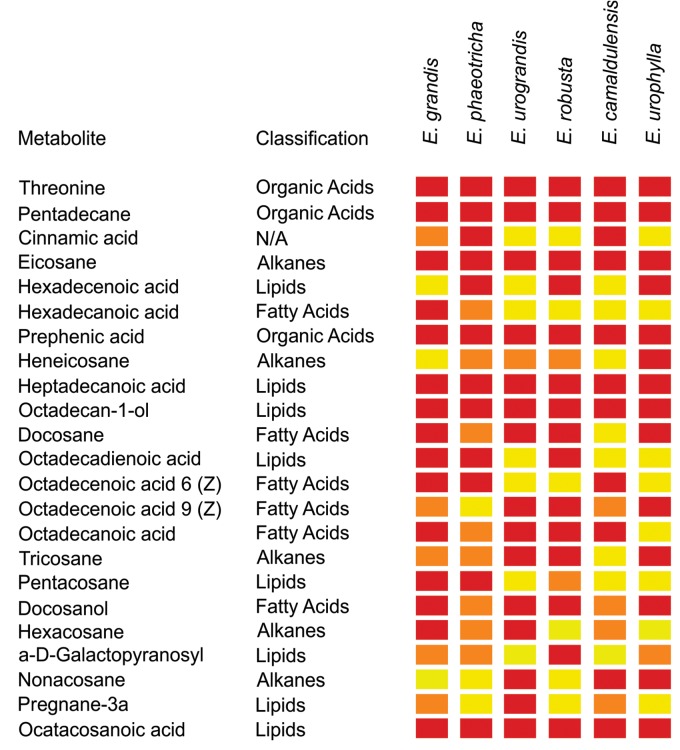
Abundance of metabolites present in the cuticle wax from susceptible (*Eucalyptus grandis* and *Eucalyptus phaeotricha*) and resistant (*Eucalyptus urograndis*, *Eucalyptus robusta*, *Eucalyptus camaldulensis* and *Eucalyptus urophylla*) *Eucalyptus* species. The data were submitted to an ANOVA followed by the Scott-Knott averages comparison test (*p* < 0.05) using the program Sisvar (v. 5. 6). Red color: indicates greater abundance of the metabolites between *Eucalyptus* spp.; orange color: less abundance of the metabolites between species, and the yellow color: indicates the absence of the metabolites in plants.

The cuticular wax compounds were classified as fatty acyls (38%), alkanes (23%), steroids (12%), organooxygen compounds (11%), hydrocarbons (4%), keto acids (4%), carboxylic acids (4%) and cinnamic acids (4%). Twenty-three compounds identified in this study have been previously described to be components of plant cuticular waxes (Table 3). The heat map obtained shows that among the identified metabolites previously described as cuticular wax compounds, hexadecanoic acid is present only in *Eucalyptus grandis* and *Eucalyptus phaeotricha* and absent in the resistant species (Figure 3).

**Table 3:** The classification of 26 metabolites presents in cuticular wax *Eucalyptus* species according of The Human Metabolome Database.

Metabolite	Class^a^	Organism	Reference
Alpha-D-Galactopyranosyl-(1,4)	Organooxygen compounds	Plant	Horbowicz et al., 1998
Androst-4-en-3,17-dione	Steroids and Steroid derivatives	Fungal	Faramarzi et al., 2008
Cinnamic acid, 4-hydroxy	Cinnamic acids and derivatives	Plant	Freitas et al., 2016
Corticosterone	Steroids and Steroid derivatives	Animal	Samtani and Jusko, 2007
Docosan-1-ol	Fatty Acyls	Plant	Stashenki and Martínez, 2012
Docosane	Alkanes	Plant	Lytovchenko et al., 2009
Eicosane	Alkanes	Plant	Asha et al., 2017
Erythronic acid	Organooxygen compounds	Human	Nikolova et al., 2016
Heneicosane	Alkanes	Plant	Bini, 2016; Moussa and Almaghrabi, 2016
Heptadecanoic acid	Fatty Acyls	Plant	Moussa and Almaghrabi, 2016
Hexacosane	Alkanes	Plant	Kumar et al., 2017
Hexadecanoic acid	Fatty Acyls	Plant	Moussa and Almaghrabi, 2016
Hexadecenoic acid	Fatty Acyls	Plant	Cahoon et al., 1994; Moussa and Almaghrabi, 2016
Nonacosane	Alkanes	Plant	Lytovchenko et al., 2009
Octacosanoic acid	Fatty Acyls	Plant	Lytovchenko et al., 2009
Octadecadienoic acid	Fatty Acyls	Plant	Bini, 2016; Moussa and Almaghrabi, 2016
Octadecan-1-ol	Fatty Acyls	Plant	Lytovchenko et al., 2009
Octadecanoic acid	Fatty Acyls	Plant	Moussa and Almaghrabi, 2016; Lytovchenko et al., 2009
Octadecenoic acid, 6-(Z)	Fatty Acyls	Plant	Rajeswari and Rani, 2015; Vijisaral and Arumugam, 2014
Octadecenoic acid, 9-(Z)	Fatty Acyls	Plant	Moussa and Almaghrabi, 2016
Pentacosane	Organooxygen compounds	Plant	Kumar et al., 2017
Pentadecane	Saturated hydrocarbons	Plant	Lytovchenko et al., 2009
Pregnane-3alpha	Steroids and Steroid derivatives	Plant	Cioffi et al., 2006
Prephenic acid	Keto acids and derivatives	Plant	Bini, 2016
Threonine	Carboxylic acids and derivatives	Plant	Bini, 2016
Tricosane	Alkanes	Plant	Casuga et al., 2016


*^*a*^The class of metabolites was determinate in The Human Metabolome Database. Access in May 2, 2018. Available in: http://www.hmdb.ca/.*

### Germination Rate of *A. psidii* in Cuticle Extracts and Hexadecanoic Acid

**FIGURE 4:**
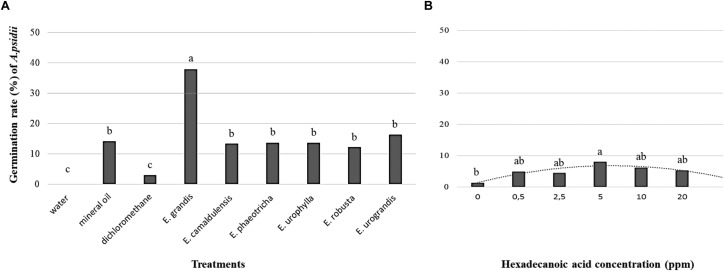
The germination rate of the *A. psidii* uredospores (MF1) at 24 h.p.i **(A)** Agar-water medium with or without cuticular extract from *Eucalyptus* species. **(B)** Agar-water medium with palmitic acid (hexadecanoic acid). The data with equal letters did not differ statistically (*p* < 0.05) using the Scott-Knott **(A)** and Tukey **(B)** averages comparison test.

The germination rate of *A. psidii* was significantly higher in the media supplemented with 20 ppm cuticle extract of *Eucalyptus grandis* (44.06%) followed by *Eucalyptus urograndis* (16.26%). With the exception of *Eucalyptus grandis*, the addition of cuticular wax from *Eucalyptus* spp. did not seem to influence the germination rates of the uredospores compared to mineral oil. Non-significant germination was observed using only the solvent dichloromethane and water (Figure 4A). To confirm the ability of hexadecanoic acid to induce germination, the germination rate of *A. psidii* was determined at 24 h.p.i. We observed the highest germination rate of 8% using 5.0 ppm hexadecanoic acid. The data showed a polynomial curve from 0 to 20 ppm. The lowest rate of 1.46% was observed in the absence of hexadecanoic acid (Figure 4B).

### Epicuticular Wax Morphology and *A. psidii* Germination *in vivo*

**FIGURE 5:**
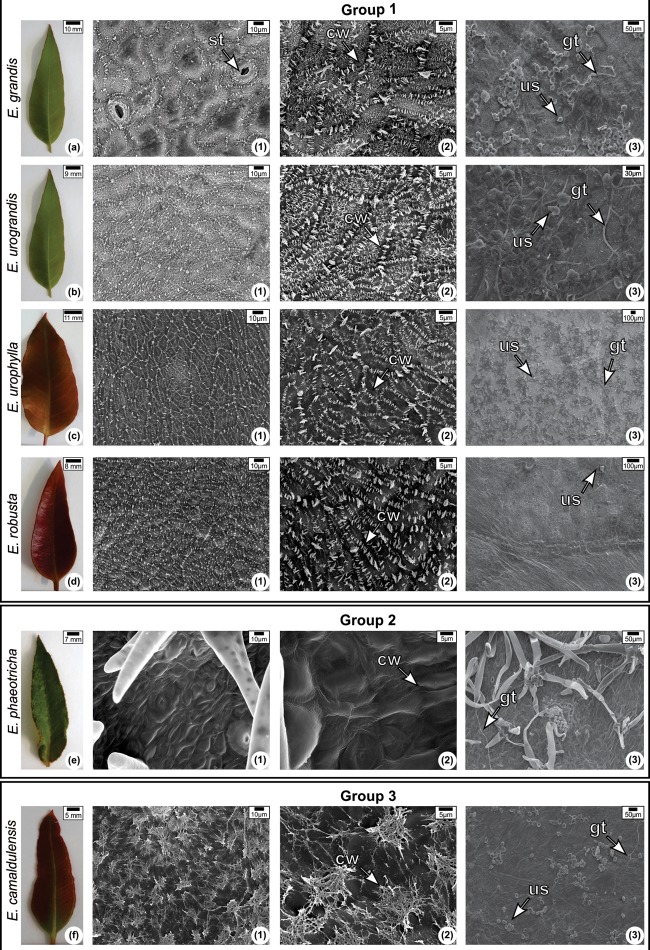
Leaf morphology **(a–f)** and scanning electron micrograph of the adaxial surface of the leaves of *Eucalyptus* species non-inoculated (1–2) and inoculated (3) with *Austropuccinia psidii* at 144 h.a.i Epicuticular wax morphology: Group I—platelets; Group II—Wax sheet; Group III—Tubes or threads. (1) and (2) were observed using different magnifications (1,100× and 3,000×, respectively). CW, cuticular wax; ST, stomata; GT, germination tube; US, Uredospore.

It was possible to group the six *Eucalyptus* spp. into three different groups based on the epicuticular wax morphology: Group I is comprised of species that contain parallel platelets wax crystals and include *Eucalyptus grandis*, *Eucalyptus urograndis*, and *Eucalyptus robusta* and *Eucalyptus urophylla* (Figures 5a–d). Group II includes *Eucalyptus phaeotricha* in which the wax crystals are absent, and the cuticle presents a smooth surface comprised of a thin wax film that can be folded in some regions (Figure 5e). Group III includes *Eucalyptus camaldulensis* and exhibits epicuticular wax distributed as tubes or threads (Figure 5f). Another difference among them is that only *Eucalyptus phaeotricha* possess a pubescent leaf covered by non-glandular trichomes (data not shown).

There are some differences in the members of Group 1 in the orientation and size of the platelets. *Eucalyptus grandis* and *Eucalyptus urograndis* possess epicuticular wax crystals of different sizes (Figures 5a1–2, b1–2). The large wax crystals are primarily located on the epidermis anticlinal walls, while the smaller ones are on the periclinal walls. In *Eucalyptus robusta*, the majority of the wax plates were parallel and arranged perpendicularly to the proximodistal leaf axis (Figures 5d1–2). *Eucalyptus urophylla* possesses parallel wax plates, but they seem to be distributed in a different direction (Figures 5c1–2).

We found germinated uredospores in all species at 24 h.p.i (data not shown). In the susceptible species *Eucalyptus grandis* and *Eucalyptus phaeotricha*, germ tubes were observed at 144 h.p.i. along with the possible presence of a degraded cuticle represented by grooves (Figure 5a3). Pustules and a substantial amount of trichomes were only observed in *Eucalyptus phaeotricha* (Figure 5e3). In *Eucalyptus urograndis*, described as a low resistance species, we also observed germinated uredospores (Figure 5b3). However, the germination was visually higher than the germinated uredospores found in *Eucalyptus urophylla* and *Eucalyptus camaldulensis* (Figures 5c3–F3). Non-germinated uredospores were found in *Eucalyptus robusta* (Figure 5d3).

## Discussion

The susceptibility level to *A. psidii* and the ID of *Eucalyptus* spp. revealed high variability among the species, as well as individuals within the same species. The susceptibility and resistance levels found in *Eucalyptus grandis*, *Eucalyptus cloeziana*, *Eucalyptus microcorys*, *Eucalyptus robusta*, *Eucalyptus tereticornis*, *Eucalyptus urograndis*, *Eucalyptus saligna* and *Eucalyptus urophylla* have been reported previously (Carvalho et al., 1998; Zauza et al., 2010; Carnegie and Lidbetter, 2012; Silva et al., 2014). However, we observed low resistance in *Eucalyptus cloeziana*, *Eucalyptus dunii* and *Eucalyptus microcorys* that had been previously described as species susceptible to *A. psidii* (Dianese et al., 1984; Kawanishi et al., 2009; Zauza et al., 2010; Carnegie and Lidbetter, 2012; Silva et al., 2014). Our research is the first report that describes the susceptibility of *Eucalyptus botryoides* and *Eucalyptus deglupta* to rust, as well as the resistance of *Eucalyptus brassiana*, *Eucalyptus exserta* and *Eucalyptus toreliodora* to the disease.

The variability within the *Eucalyptus* species has resulted in a controversial susceptibility classification of *A. psidii*, which could be explained by the high level of allogamy found in *Eucalyptus*. The pathogen *A. psidii* has also been described to possess a wide genetic variability (Quecine et al., 2014). In addition, it is known that the incidence and severity of the disease varies among genotypes within the same species, geographic region and time of the year (Alfenas et al., 2004), demonstrating the importance of the genotype–environment interaction on myrtle rust studies.

In controlled conditions, we validated the data obtained in the field. The assay demonstrated the ability of the uredospores from *Eucalyptus grandis* to infect the susceptible species *Eucalyptus phaeotrica*. In addition, the symptoms of the disease were visualized early in *Eucalyptus phaeotrica* when compared to *Eucalyptus grandis*. At 336 h.p.i., *Eucalyptus camaldulensis* and *Eucalyptus robusta* showed a hypersensitive response, which was not observed in *Eucalyptus urophylla* and *Eucalyptus urograndis*. The different defense responses exhibited in the species evaluated against *A. psidii* reflect the enormous complexity in the plant responses. The SYBR Green-based qPCR assay was used for the first time to detect *A. psidii* on six different *Eucalyptus* species in initial infection times, validating the sensitivity of the set IGS7/IGS9 primers used to quantify the rust pathogen in *Eucalyptus grandis* (Bini et al., 2018).

The qPCR corroborated the field results. Temporal monitoring of *A. psidii* showed its presence in a very low abundance until 72 h.p.i in all the species, independently of their susceptibly level. After 72 h.p.i., *A. psidii* was found only in *Eucalyptus grandis* and *Eucalyptus phaeotricha* (susceptible species). Our data is consistent with that of Xavier et al. (2001) and Leite (2012). The authors compared the *A. psidii* infection process in two contrasting genotypes of *Eucalyptus grandis* and verified that fungal germination, appressoria formation and penetration occur within 12 h.p.i., independent of the genotype. The defense response starts in the resistant species at 24 h.p.i. After 72 h.p.i, is not possible to detect the pathogen in the resistant hosts, while in the susceptible plants, a succession of events occurs, including mesophyll colonization, development and sporulation pustules. The success of the *A. psidii* x *Eucalyptus* interaction is primarily defined in the first stage of infection, and the preformed mechanisms probably play an important role in resistance. Further, the qPCR assay to quantify *A. psidii* should be proceed with other *Eucalyptus* species.

GC-TOF-MS identified 26 compounds from the cuticular waxes of *Eucalyptus* leaves. Twenty-three compounds were plant specific, belonging to different classes, such as fatty acyls, alkanes, steroids and hydrocarbon carboxylic acids. Commonly, the compounds of cuticular waxes are derived from very long chain fatty acids (VLCFA), including alkanes, alcohols, and sterols as found in this study (Racovita et al., 2015). The fatty acids in the cuticles of plants and insects have significant effects on spore germination and fungal differentiation, and may be toxic, fungistatic and stimulatory for some pathogenic species. For example, Golebiowski et al. (2008) obtained the profiles of cuticular fatty acids of three species (*Calliphora vicina*, *Dendrolimus pini* and *Galleria mellonella*) in relation to the susceptibility to infection caused by *Conidiobolus coronatus*. The species resistant to *C. vicina* had a different lipid profile compared to the susceptible species *D. pini* and *G. mellonella*. The exclusive presence of three fatty acids in the cuticle of the resistant genotype could inhibit fungal growth and reduce the production of conidia. Our data revealed differences in the lipid profile among *Eucalyptus* spp. resistant and susceptible to *A. psidii* in a manner consistent with these results.

We found a specific fatty acid, hexadecanoid acid, in susceptible *Eucalyptus grandis* and *Eucalyptus phaeotricha* that improved the uredospore germination rates of *A. psidii*. The role of this compound in the biosynthesis of cuticular wax is highly diverse. Hexadecanoic acid participates in the biosynthetic pathways of fatty acids, cutin, suberin, wax, unsaturated fatty acids and secondary metabolites from plants and contributes to the elongation and degradation of fatty acids (Kanehisa and Goto, 2000).

Unexpectedly, only the cuticle waxes from *Eucalyptus grandis* stimulated the germination of *A. psidii* uredospores, and the germination rate in *Eucalyptus phaeotricha* did not differ from the control. Our data is supported by other research, such as that of Song-Jiang et al. (2014) who used cuticular waxes from pingguoli pear to stimulate the germination and mycelial growth of *Alternaria alternata*. Another study showed that an epicuticular wax extract of wheat leaf (*Triticum compactum* L.) had an active component capable of inducing up to 50% the formation of the germ tube, appressorium, substomatal vesicle and penetrating hyphae of *Puccinia graminis* f.sp. *tritici* (Reisige et al., 2006). These data enhance the importance of cuticular wax as physical and chemical signaling patterns in the recognition of the host by the pathogen.

Tessmann and Dianese (2002) observed that a long-chain hydrocarbon with 436 MW (C31H64), hentriacontane, obtained from *S. jambos* leaf wax extract could induce uredospore germination up to 88% in *A. psidii*. We observed that the effect of hexadecanoic acid on the *A. psidii* germination rate was lower than in the media supplemented with *Eucalyptus* cuticular waxes. However, our data confirm the influence of hexadecanoic acid on the susceptibility of *Eucalyptus* spp. to the pathogen *A. psidii* and suggest a complex *A. psidii* x *Eucalyptus* spp. interaction related to probable physical and chemical signaling.

Our data also revealed global differences in the lipid profile between *Eucalyptus* spp. resistant and susceptible to *A. psidii*, and not only the chemical profile of possible preformed mechanism related to resistance in *Eucalyptus* spp. was evaluated. It was possible to assemble *Eucalyptus* spp. in three groups based on their epicuticular wax morphologies. In the s, Hallam and Chambers (1970) undertook a large study to characterize the wax morphology from a survey of 315 *Eucalyptus* species and classified them in groups. These authors found that *Eucalyptus camaldulensis* has the most plastic epicuticular wax. In this study, we observed that *Eucalyptus camaldulensis* has tubes or threads-shaped epicuticular wax that resemble the *Eucalyptus globulus* wax pattern (Steinbauer et al., 2009), but this was differed from the observations of Guzmán et al. (2014) who described the epicuticular wax arrangements as plates with different orientations. Previous studies demonstrated that topography is important for the formation of appressoria (Read et al., 1992; Marques et al., 2013). We believed in a correlation of epicuticular morphology as a determining factor to *Eucalyptus* susceptibility or resistance. However, this characteristic is better related with the cuticular wax chemical composition. It is clear that *A. psidii* infects species with different cuticular morphologies suggesting that the morphology it is not the key factor for susceptibility.

*Eucalyptus* spp. have more than one mechanism (preformed and induced resistance) responsible for their resistance against *A. psidii*. The cuticular chemical composition is strongly related to the susceptibility of *Eucalyptus grandis* and *Eucalyptus phaeotricha*. However, our data suggest that there are many cuticular signals that act at different stages of fungal infection, uredospore germination, appressorium formation, invasion and survival on the eucalyptus leaves, corroborating the hypothesis that the *Eucalyptus* spp. resistance to *A. psidii* is related to synergistic preformed and induced resistance mechanisms that should be studied in more detail.

## Author Contributions

IS and MQ designed the research. IS, ML, AB, BT, EF, TC, JM, and LS performed the experiments. IS, TC, JM, and BV analyzed the data. IS, MQ, TC, JM, and CL wrote and revised the manuscript. IS, MQ, JM, and CL contributed through discussions.

## Conflict of Interest Statement

The authors declare that the research was conducted in the absence of any commercial or financial relationships that could be construed as a potential conflict of interest.

## Footnotes

1 http://gmd.mpimp-golm.mpg.de/

2 http://www.metaboanalyst.ca/
